# Oxidative Stress as a Physiological Pain Response in Full-Term Newborns

**DOI:** 10.1155/2017/3759287

**Published:** 2017-01-04

**Authors:** S. Perrone, C. V. Bellieni, S. Negro, M. Longini, A. Santacroce, M. L. Tataranno, F. Bazzini, E. Belvisi, A. Picardi, F. Proietti, L. Iantorno, G. Buonocore

**Affiliations:** Department of Molecular and Developmental Medicine, University of Siena, 53100 Siena, Italy

## Abstract

This research paper aims to investigate if oxidative stress biomarkers increase after a painful procedure in term newborns and if nonpharmacological approaches, or sex, influence pain degree, and the subsequent OS. 83 healthy term newborns were enrolled to receive 10% oral glucose or sensorial saturation (SS) for analgesia during heel prick (HP). The ABC scale was used to score the pain. Advanced oxidation protein products (AOPP) and total hydroperoxides (TH) as biomarkers of OS were measured at the beginning (early-sample) and at the end (late-sample) of HP. The early-sample/late-sample ratio for AOPP and TH was used to evaluate the increase in OS biomarkers after HP. Higher levels of both AOPP and TH ratio were observed in high degree pain (4–6) compared with low degree pain score (0–3) (AOPP: *p* = 0.049; TH: *p* = 0.001). Newborns receiving SS showed a significantly lower pain score (*p* = 0.000) and AOPP ratio levels (*p* = 0.021) than those without. Males showed higher TH levels at the end of HP (*p* = 0.005) compared to females. The current study demonstrates that a relationship between pain degree and OS exists in healthy full-term newborns. The amount of OS is gender related, being higher in males. SS reduces pain score together with pain-related OS in the newborns.

## 1. Introduction

Nociceptive pathways are working from an early stage of fetal development [1]. Indeed, neonates have functional ascending (excitatory) pain pathways by 24 weeks' gestation, but descending (inhibitory) pathways appear immature until approximately 48 weeks' gestation [2, 3]. Here, the importance of reducing neonatal pain is discussed. Neonates have also many other immature pain responses, which expose them to a greater intensity of pain for a prolonged period of time [4]. Repeated invasive procedures occur routinely in neonates who require intensive care [5]. Therefore, ill full-term newborns and preterm infants are prone to significant painful stimuli at a time when the developing nervous system is notoriously sensitive to changes in sensory experience [6]. There is increasing clinical evidence that painful stimulations elicit specific behaviors, activate the somatosensory cortex, and stimulate neuroendocrine and physiological stress responses, thus leading to short- and long-term clinical consequences in newborns [7–10]. In preterm newborns, repeated and prolonged pain exposures alter their subsequent pain modulation, pain reactivity, neurodevelopment, and behavior [11, 12]. Full-term born children, who needed neonatal intensive care, showed enhanced perceptual sensitization to prolonged painful stimulation and hypoalgesia to brief heat pain stimuli at the age of 9–14 years [13]. On healthy full-term infants, only few early studies suggest an alteration in pain threshold because of pain experience [14–16]. Intercurrent illness, duration of care, and gestational age are some confounding factors that may alter the real role of painful stimuli or tissue damage in clinical cohorts [17]. Moreover, all comorbidities, different scales for pain assessment, and wide pharmacological and nonpharmacological approaches decrease the specificity of behavioral and physiological response to pain [18]. Therefore, identifying underlying mechanisms of pain-related consequences, along with reliable biomarkers to combine with pain scales, is of paramount importance for future researches.

A recent study in preterm babies showed a significant correlation between biomarkers of oxidative stress (OS) and pain score after tape removal during discontinuation of an indwelling central arterial or venous catheter [19]. Previously, Bellieni et al. demonstrated the same correlation also during a heel prick (HP) procedure [20]. Little is known about free radicals (FR) generation after painful procedures in newborns. Conversely, a lot of evidence reports the FR toxicity in the neonatal period [21]. The current study tested the hypothesis that OS could be elicited also in healthy full-term infants after their first HP. The correlation between OS, pain score, and grade was also tested. Our secondary aim was to analyze the variations in pain-related OS level between two different nonpharmacological approaches and its relation to newborns' sex.

## 2. Materials and Methods

A prospective research study was performed at the University Hospital of Siena between January 2014 and June 2014. All healthy term newborns with normal adaptation to extrauterine life, who underwent a routine HP to perform blood sampling for metabolic screening at 48 hours of life, were consecutively enrolled in the study. Exclusion criteria were congenital anomalies, chromosomopathies, newborns from mothers with drug abuse or any systemic analgesic treatment, night time samplings, newborns with any previous HP, need of multiple HP, and nonsufficient blood collection for a proper analysis. 83 newborn were included in the study (see Table 1 for details). Study protocol was approved by the local ethic board. An informed consent was collected from the parents before the enrollment.

After informed consent was obtained, newborns were consecutively enrolled and by lottery included into two different groups. The first group received 1 mL of oral 10% glucose solution (10% OG) before and during HP, a midwife instilled the solution in the newborn's mouth, and the sucking was induced with a finger enveloped by a glove. The second group received the sensorial saturation (SS) [22], in which mothers simultaneously attract their infant's attention by massaging the cheek, speaking to the baby gently, and administering 1 mL of 10% OG, the latter similarly to the previous group. The same assistant, who gave standardized information, trained each mother to the technique at least 4 hours before the sampling. In all cases in which mothers refused to perform the procedure at the time of the sampling (e.g., fear for the HP, stress feeling, impossibility to cope with sampling at the right time, or insecurity) 10% OG solution was administered and the infants were switched to the first group. The final population consisted of sixty-nine babies receiving 1 mL of 10% OG solution and fourteen babies receiving SS for analgesia.

### 2.1. Test Procedure

The sampling procedure was divided into 3 phases: (1) after heel cleansing and the prick, 100 *μ*L of blood for the early OS measurements in a microtube (early-sample) was collected; (2) 12 drops of blood for the routine metabolic screening were taken; (3) 100 *μ*L of blood for the late OS measurements in a microtube (late-sample) was collected. The average time that elapsed from the beginning to the end of the HP procedure was 1 minute and 30 seconds.

Immediately after the HP (during phase 1), pain was scored using the ABC pain scale by an independent assistant who was blinded to the study. This assistant received training for ABC pain scale use before the beginning of the study. To minimize bias due to interscore variability, a single assistant was used. The ABC scale is a validated and an easy to use pain scale to establish the grade of acute pain in both term and preterm babies [23]; it estimates the pitch of the first cry, the crying rhythmicity, and the constancy of the crying during the first 30 seconds after a definite painful stimuli; it ranges from no pain (ABC score = 0) to maximum pain (ABC score = 6) (see Table 2). This scale does not detect the effect of the squeezing.

Early- and late-samples were stored and analyzed to determine protein and lipid OS-induced injury by measuring blood levels of advanced oxidation protein products (AOPP) and total hydroperoxides (TH). These biomarkers provide information about the level of OS in newborns [21, 24]. In particular, TH are the intermediate oxidative products of lipids, protein, and amino acids, and therefore they represent a measure of overall OS. TH concentrations were evaluated using a d-ROMs Kit (Diacron srl, Italy) [24]. AOPP reflect oxidized plasma proteins and their concentrations were detected using spectrophotometry on a microplate reader. The changes (ratio) between the two time points for AOPP and TH were calculated to evaluate the increase in OS biomarkers before and after the HP.

### 2.2. Statistical Analysis

Analyses were performed using the SPSS v20 for Windows statistical package (SPSS Inc., Chicago, IL, USA). The paired *t*-test was used to verify the difference in the ABC pain score and in OS biomarkers between the two time points, while the unpaired *t*-test or the Mann–Whitney test was used, as appropriate, to evaluate pain in relation to gender, analgesia, and OS biomarkers. The Pearson test was used to verify the existence of a linear correlation between the pain ABC score and OS biomarkers levels.

## 3. Results

The descriptive analysis of the population is reported in Table 1.

Before the procedure, all the newborns were quiet and relaxed (mean ABC score before HP: 0.00 ± 0.00) and a significant increase in the ABC pain score was observed after HP (mean ABC score: 2.66 ± 1.81; *p* = 0.000). According to the type of analgesia, a significant decrease of the ABC pain score was found in infants treated with SS compared to that in those with 10% OG (respectively: 0.42 ± 0.85 versus 3.11 ± 1.61; *p* < 0.0001).

### 3.1. Pain-Related Oxidative Stress Results

**Figure 1:**
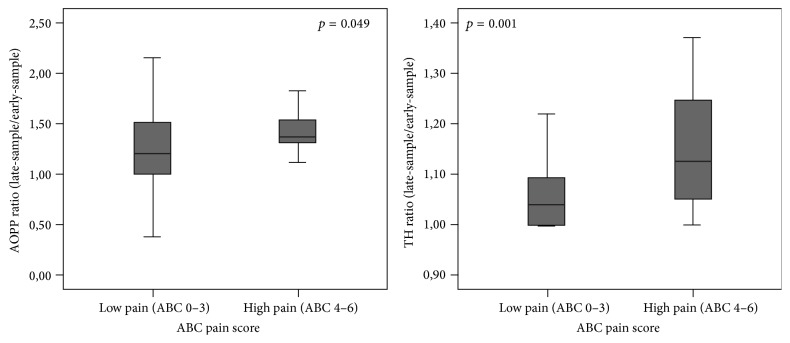
Mean of AOPP and TH ratio (late-sample/early-sample) depending on ABC pain scores.

**Figure 2:**
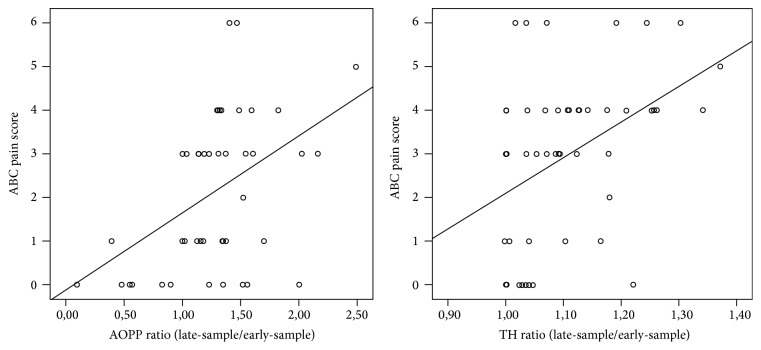
Linear correlation between OS biomarkers (AOPP and TH ratio, late-sample/early-sample) and ABC pain scores.

After the HP AOPP blood levels significantly increased (AOPP, early-sample: 34.44 ± 16.70 versus AOPP late-sample: 42.91 ± 27.71; *p* = 0.007), while TH blood levels increased but not significantly (TH early-sample: 244.99 ± 85.16 versus TH late-sample: 257.07 ± 85.89; *p* = 0.058). Evaluating OS according to pain score, significantly higher levels of both AOPP and TH ratio were observed in high levels of pain (4–6) compared to low levels of pain (0–3) (resp., AOPP ratio 1.49 ± 0.35 versus 1.20 ± 0.45  *p* = 0.049; TH ratio 1.14 ± 0.11 versus 1.05 ± 0.06, *p* = 0.001, Figure 1). A significant linear correlation was found between pain scores and AOPP ratio and TH ratio (resp., *r* = 0.453, *p* = 0.002; *r* = 0.423, *p* = 0.002, Figure 2).

**Figure 3:**
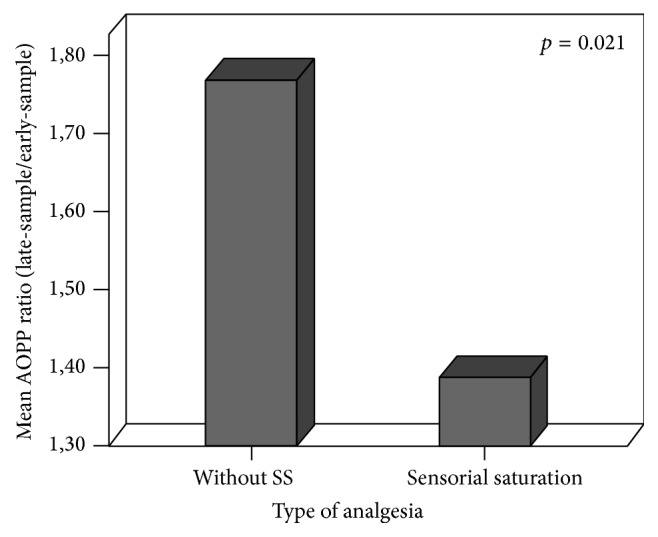
Mean of AOPP ratio (late-sample/early-sample) depending on type of analgesia.

Significantly decreased AOPP ratio levels were also observed in newborns treated with SS compared to those without (resp., 1.38 ± 0.87 versus 1.76 ± 1.13, *p* = 0.021, Figure 3). No differences in TH ratio levels were found according to the type of analgesia (TH ratio in SS: 1.45 ± 1.93 versus TH ratio in 10% OG: 1.69 ± 2.30, *p* = 0.694).

### 3.2. Sex-Related Results

**Figure 4:**
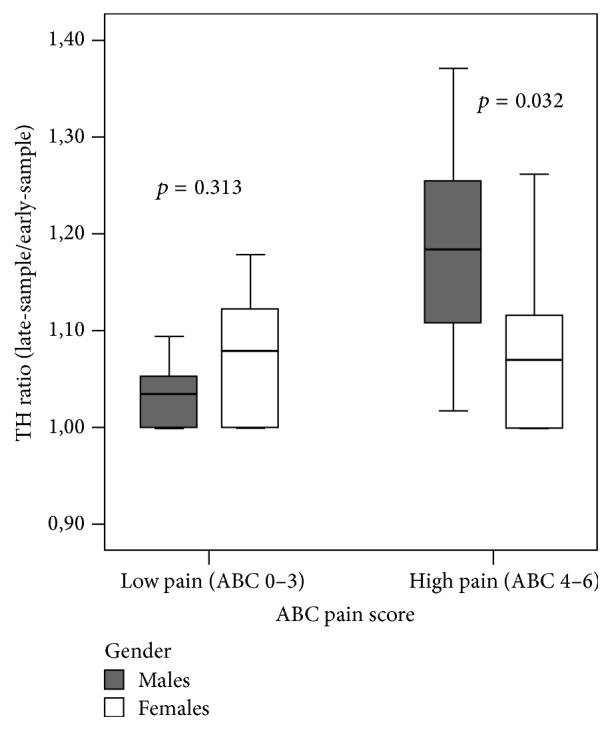
Mean values of TH ratio according to the level of pain, reported by gender.

**Figure 5:**
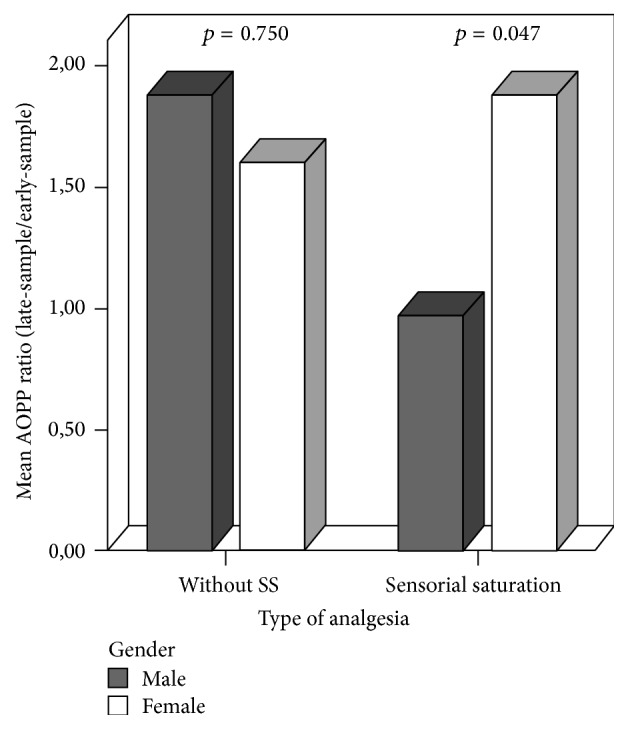
Gender differences in mean of AOPP ratio (late-sample/early-sample) depending on type of analgesia.

No difference in the ABC pain score was observed between males and females (resp., 2.60 ± 1.89 versus 2.75 ± 1.71; *p* = 0.701), independently of the type of analgesia. No sex differences were found in AOPP levels measured at the beginning and at the end of HP procedures. Males showed significant higher lipid peroxidation than females at the end of the HP (TH late-sample, resp., 277.50 ± 85.66 versus 222.76 ± 73.71, *p* = 0.005), which was also confirmed for higher degrees of pain (TH ratio, M: 1.18 ± 0.10 versus F: 1.07 ± 0.08, *p* = 0.032, Figure 4). The linear correlation existing between ABC pain score and OS markers reported by newborn's sex persisted only for males (AOPP ratio: *r* = 0.583, *p* = 0.003; TH ratio: *r* = 0.506, *p* = 0.003). AOPP ratio was significantly lower in males than in females after SS administration (0.96 ± 0.66 versus 1.87 ± 0.56, *p* = 0.047, Figure 5).

## 4. Discussion

The results of the present study were in agreement with the current literature that reports an association between a routine painful procedure and the subsequent FR production in infants [19, 20]. This work also showed a linear correlation between OS biomarker levels and pain score. Slater et al. have found the same evidences using a different pain procedure (tape removal during discontinuation of an indwelling central catheter), biomarker (malondialdehyde), and pain scale (Premature Infant Pain Profile scale) [19], thus giving strength to the present finding. For the first time in literature, this pain-related stress response was evaluated in healthy full-term infants with no other pain experiences or confounding factors related to critically ill conditions or prematurity.

We believe that this physiologic reaction may play a crucial role in pain-related stress consequences. Many conditions of increased FR release occur in ill term babies who require intensive care and some long-term pain-related consequences were found also in these cohorts [13]. In healthy term infants, the hyperoxic challenge between intra- and extrauterine environment represents the main FR source and OS can consequently occur [25]. No data is available about term newborn's susceptibility to isolated increase in OS level after 48 hrs of life and literature lacks of follow-up studies in this kind of population. However, their less impaired antioxidant defenses and the rarity of the insults may generate an easier and faster neutralization of FR, thus preventing long-term consequences. Anyhow, FR production occurs soon after a painful stimulus also in term babies; thus OS could be finally proposed as an independent physiological response to pain-related stress or tissue damage. We believe that glutamatergic stimuli can play a pivotal role in this correlation, but further studies are needed to deeply understand the underlying mechanisms. The significant increase only in AOPP blood concentrations following pain stimuli may reflect the role of proteins as the first line defense against oxidative injury, as previously suggested by our research group [26]. The present study also supports our previous findings showing a clear association between high pain degree and higher OS levels in newborns [20]. The latter association was found for both of the tested biomarkers; thus lipid and protein peroxidation could be involved during a stronger pain experience, but there are no data in literature supporting this theory.

In this study, a routine HP procedure causes pain-related stress in newborns, in both males and females. However, according to pain score, the entity of OS was sex-related, being higher in males than females. Males showed higher lipid peroxidation than females not only at the end of the prick, but also when the grade of pain was high. It was previously demonstrated that estrogen activates the membrane associated estrogen G protein-coupled receptor (GPR30), which influence the signaling cascades of opioid receptors. Therefore, sex differences in pain-related OS biomarkers level may be due to the organizational effects of steroids during a critical period of development [27]. The protective estrogen's effect against OS is also shown by several in vitro and in vivo experiments. Giordano et al. demonstrated in a recent animal study that estrogen modulates the cerebral expression of some antioxidant enzymes (paraoxonase 1 and paraoxonase 2, PON1 and PON2), thus increasing the resistance of female neurons to OS damage [28]. Though estrogen's neuroprotective effects are well known [29, 30], the absence of the estradiol's protective effect on cells from PON2 knockout (PON2^−/−^) mice suggests that a major mechanism of estrogen-related neuroprotection may be represented by the induction of a specific antioxidant enzyme [28]. Gender disparities in OS levels and their relationship with the hormonal status were also underlined in another study from Minghetti et al. in which males showed higher lipid peroxidation and lower antioxidant capacity than females, contributing to a better understanding of the male disadvantage in OS injury [31]. The exact mechanisms through which these sex-related differences act are still largely unknown, but differential genotypes [32] and the disparity in the operation of the gonadal hormones- and opioid-modulating-pain circuits may be an explanation [33]. The downstream mechanisms involve cytosolic calcium increase [34], protein kinase A, protein kinase B, protein kinase C, phospholipase C, inositol triphosphate activation, neuronal membrane depolarization, and ROS accumulation [35].

In a previous work, we stated that SS is a noninvasive, easily performed, and reproducible technique for a complete analgesia during HP in neonates [36]. The present study showed that SS significantly reduces pain and its subsequent pain-related OS in full-term newborns, with significant lower AOPP levels in males than females. The evidence that a proper analgesic procedure could protect patients from pain-related OS is one of the main findings of our paper as this result may represent one of the most important future perspectives in this field. Unfortunately, the small sample size of SS avoids any kind of conclusion in this sense and further studies are required to confirm these data.

The present study has some limitations. First of all, the SS group number was small. Nevertheless, the disparity in numbers between groups did not affect the statistical power of the study. Second, the examiner was not blinded to the patient group, but since a complete blinded examination was impossible due to the visual difference between the two procedures, we involved a collaborator who was present during the procedure but not aware of the aims of the study, in order to limit the bias. Third, the choice of 10% oral glucose followed our previous study design in premature babies [20], in order to use a well-known tool that is also validated by the Italian Society of Neonatology guidelines [37]. There are some evidences that sweet solutions less than 18% are ineffective to reduce infant pain optimally [38]. Otherwise, studies conducted by Bellieni et al. reported the use of 10% OG as an effective analgesic treatment when associated with nonnutritive sucking [39]. We believe that research studies should be performed as homogeneously as possible; of course, with more concentrated glucose solutions for analgesia the average level of pain might be different but less physiologic and most babies would display a wide variety of responses.

## 5. Conclusions

For the first time in literature, the current study demonstrates that a relationship between degree of pain and OS exists also in healthy full-term newborns at their first pain exposure. This evidence proposes a new insight into pain-related responses and contributes to suggesting a potential novel mechanism of damage after repeated and undermanaged painful experiences. The severity of OS injury is sex-related, higher in males than in females, thus indicating an innate susceptibility of males to OS. SS seems to be a promising procedure to reduce not only pain but also pain-related OS in newborns, giving chance to focusing on future researches in this field. Nevertheless more studies and a bigger sample size are needed to confirm our data.

## Figures and Tables

**Table 1:** Descriptive analysis of the population, reported by groups.

	10% oral glucose (*n* = 69)	Sensorial saturation (*n* = 14)	*p*
GA (weeks) mean (SD)	38 (1)	39 (1)	ns
BW (g) mean (SD)	3209 (477)	3365 (328)	ns
Gender absolute frequencies (%)			
M	41 (59.4)	9 (64.3)	ns
F	28 (40.6)	5 (35.7)	
AOPP presample (umol/l) mean (SD)	59.73 (49.50)	43.71 (37.21)	ns
TH presample (UCARR/l) mean (SD)	250.89 (107.48)	277.87 (78.09)	ns
ABC score at the beginning of heel prick median (IQ)	0 (0)	0 (0)	ns

**Table 2:** ABC scale.

Parameters	Score	
Acuteness of the first cry	Absent	0
	Present	2

Burst rhythmicity	Absent	0
	Present	2

Constancy of the crying intensity	Absent	0
	Intermediate	1
	Constant	2

## Abbreviations

10% OG: 10% oral glucose solution
AOPP: Advanced oxidation protein products
FR: Free radicals
GPR30: G protein-coupled receptor
HP: Heel prick
OS: Oxidative stress
PON: Paraoxonase
PON2^−/−^: Paraoxonase 2 knockout mice
SS: Sensorial saturation
TH: Total hydroperoxide.
